# Diagnosing early-onset neonatal sepsis in low-resource settings: development of a multivariable prediction model

**DOI:** 10.1136/archdischild-2022-325158

**Published:** 2023-04-27

**Authors:** Samuel R Neal, Felicity Fitzgerald, Simba Chimhuya, Michelle Heys, Mario Cortina-Borja, Gwendoline Chimhini

**Affiliations:** 1 Population, Policy and Practice, UCL Great Ormond Street Institute of Child Health, London, UK; 2 Infection, Immunity and Inflammation, UCL Great Ormond Street Institute of Child Health, London, UK; 3 Child and Adolescent Health Unit, University of Zimbabwe, Harare, Zimbabwe

**Keywords:** Global Health, Infectious Disease Medicine, Intensive Care Units, Neonatal, Neonatology, Sepsis

## Abstract

**Objective:**

To develop a clinical prediction model to diagnose neonatal sepsis in low-resource settings.

**Design:**

Secondary analysis of data collected by the Neotree digital health system from 1 February 2019 to 31 March 2020. We used multivariable logistic regression with candidate predictors identified from expert opinion and literature review. Missing data were imputed using multivariate imputation and model performance was evaluated in the derivation cohort.

**Setting:**

A tertiary neonatal unit at Sally Mugabe Central Hospital, Zimbabwe.

**Patients:**

We included 2628 neonates aged <72 hours, gestation ≥32^+0^ weeks and birth weight ≥1500 g.

**Interventions:**

Participants received standard care as no specific interventions were dictated by the study protocol.

**Main outcome measures:**

Clinical early-onset neonatal sepsis (within the first 72 hours of life), defined by the treating consultant neonatologist.

**Results:**

Clinical early-onset sepsis was diagnosed in 297 neonates (11%). The optimal model included eight predictors: maternal fever, offensive liquor, prolonged rupture of membranes, neonatal temperature, respiratory rate, activity, chest retractions and grunting. Receiver operating characteristic analysis gave an area under the curve of 0.74 (95% CI 0.70–0.77). For a sensitivity of 95% (92%–97%), corresponding specificity was 11% (10%–13%), positive predictive value 12% (11%–13%), negative predictive value 95% (92%–97%), positive likelihood ratio 1.1 (95% CI 1.0–1.1) and negative likelihood ratio 0.4 (95% CI 0.3–0.6).

**Conclusions:**

Our clinical prediction model achieved high sensitivity with low specificity, suggesting it may be suited to excluding early-onset sepsis. Future work will validate and update this model before considering implementation within the Neotree.

**WHAT IS ALREADY KNOWN ON THIS TOPIC**

- Neonatal sepsis is difficult to diagnose as the clinical features are non-specific.
- In low-resource settings, early neonatal care may be led by less experienced healthcare professionals without immediate local senior support.
- Clinical prediction models exist to diagnose neonatal sepsis but there is a need for models suitable to implement in low-resource settings.

**WHAT THIS STUDY ADDS**

- Our model has been specifically developed in a cohort of neonates from a lower middle-income, low-resource neonatal unit in sub-Saharan Africa.
- It is easy to implement in low-resource settings as it does not require laboratory tests.

**HOW THIS STUDY MIGHT AFFECT RESEARCH, PRACTICE OR POLICY**

- Our model predicts a diagnosis of early-onset sepsis made by an experienced neonatologist to support less experienced healthcare professionals admitting neonates to the neonatal unit.

## Introduction

Neonatal sepsis caused 15% of the 2.5 million neonatal deaths worldwide in 2018 and has a mortality rate of 110–190 per 1000 live births.^1 2^ It can be difficult to diagnose as the clinical features overlap with non-infectious diseases.^3^ Failing to treat sepsis with timely antimicrobials increases the risk of death or disability, but empirical antimicrobial therapy in non-infected neonates contributes to antimicrobial resistance and adverse outcomes.^4 5^

Isolating a pathogenic organism from a normally sterile site is the gold standard diagnostic method,^6^ but has limitations. In low-resource settings (LRS), cultures and blood counts are often unavailable,^7^ or turnaround times are too long to usefully inform management.^8 9^ Blood cultures have high sensitivity provided sufficient inoculate volume is obtained, but sampling can be difficult in unwell neonates.^10^ Therefore, clinicians may diagnose sepsis and initiate empirical therapy despite negative cultures, based on clinical presentation, risk factors and/or raised inflammatory markers. This is often called ‘culture-negative’ sepsis and up to 16 times more neonates receive antibiotics for culture-negative sepsis than for sepsis with a positive culture.^11^ Diagnostic challenges are increased in LRS where early neonatal care may be led by less experienced healthcare professionals (HCPs) without immediate local senior support.^8^

Clinical prediction models combine patient or disease characteristics to estimate the probability of a diagnosis or outcome.^12^ Models to diagnose neonatal sepsis may improve diagnostic accuracy and rationalise antibiotic use. In LRS, they could provide clinical decision support for less experienced HCPs, especially if models do not require laboratory tests. Several existing models estimate the probability of neonatal sepsis, but few are developed for LRS.^13 14^

Our objective was to develop a clinical prediction model to diagnose neonatal sepsis in an LRS neonatal unit, to support less experienced HCPs make this diagnosis.

## Methods

We report methods according to the Transparent Reporting of a Multivariable Prediction Model for Individual Prognosis or Diagnosis statement (online supplemental file 1).^15^ Further methods are found in online supplemental file 2 and accompanying R code at https://zenodo.org/record/7817247.

10.1136/archdischild-2022-325158.supp1 Supplementary data

### Source of data

We performed secondary analysis of data from the Neotree at the neonatal unit of Sally Mugabe Central Hospital (SMCH), Zimbabwe. Data were collected over 14 months from 1 February 2019 to 31 March 2020.

The Neotree is an open-source digital health system for newborn care in LRS,^16^ embedded in routine practice at three neonatal units in sub-Saharan Africa (Kamuzu Central Hospital, Malawi; SMCH, Zimbabwe; and Chinhoyi Provincial Hospital, Zimbabwe).^17^ On admission, HCPs complete an admission form using the Neotree application on an Android tablet. The application guides assessment of the neonate and collects predefined data. At discharge or after neonatal death, HCPs complete an outcome form, which includes the final diagnoses or cause(s) of death after review by a consultant neonatologist (online supplemental file 2, section 1).

### Participants

SMCH has the largest of three tertiary neonatal units in Zimbabwe, with 100 cots. Most admissions come directly from the labour ward or obstetric theatre, but SMCH is also a national referral centre for specialist surgical care.

We included neonates with chronological age <72 hours, ≥32^+0^ weeks’ gestation at birth and birth weight ≥1500 g. We excluded non-first-born multiples and those with a diagnosis of major congenital anomaly, no outcome form completed or anomalous admission durations (eg, date of discharge before date of admission).

### Outcome

The primary outcome was clinical early-onset neonatal sepsis (EOS), defined as sepsis with onset within the first 72 hours of life, as diagnosed by the treating consultant neonatologist and recorded on the outcome form as one or more of: (1) primary discharge diagnosis, (2) additional problem during admission, (3) primary cause of death or (4) contributory cause of death. No specific actions were performed to blind outcome assessment.

### Predictors

We identified candidate predictors through a modified Delphi method study^18^ and literature review.^13^ We mapped these predictors to available Neotree data, yielding 22 candidate predictors (online supplemental file 2, section 2). No specific actions were performed to blind predictor assessment.

### Statistical analysis

Analyses were performed in RStudio V.2022.02.0+443 (R V.4.1.3).^19 20^ No specific sample size calculations were performed but post hoc calculations are shown in online supplemental file 2, section 9.

#### Data preparation

We linked admission and outcome forms using the Fellegi-Sunter method of probabilistic record linkage (online supplemental file 2, section 4).^21 22^ We imputed missing values using multivariate imputation by chained equations assuming missing at random with 40 imputed data sets (online supplemental file 2, section 6).^23^

#### Model development and specification

We used multivariable logistic regression to predict diagnosis of clinical EOS. For convenience, model selection was performed in one data set randomly selected from all imputed data sets. First, we fitted a ‘full’ main effects model containing all candidate predictors assuming linearity of continuous predictors and additivity at the predictor scale. We excluded categorical variables with skewed distributions (<5% category prevalence in either outcome group) if Fisher’s exact test was non-significant (p≥0.05) for the m×n contingency table. Otherwise, skewed categorical predictors were retained, and smaller categories combined into an ‘other’ category. Next, we compared plausible variations to the full model, selecting the ‘optimal’ model which minimised both the Akaike and Bayesian information criteria (online supplemental file 2, section 8). We explored non-linear effects of continuous predictors with natural cubic spline functions (2–10 df) and polynomial transformations (second-degree to fifth-degree polynomials), and tested for interaction between birth weight and gestational age. Finally, we fitted the optimal model across all imputed data sets and obtained pooled regression coefficients and their SEs using Rubin’s rules.^24^

#### Model performance

We evaluated the performance of the optimal model in the derivation cohort. Discrimination was quantified by plotting a receiver operating characteristic curve in each imputed data set. We pooled the area under the curve (AUC) and variance across imputed data sets using Rubin’s rules.^24^ Calibration was assessed by plotting a flexible calibration curve with a loess smoother in the single data set used for model selection.^25^ Sensitivity, specificity, predictive values and likelihood ratios of the optimal model were estimated in the single data set used for model selection. These metrics are presented for the ‘optimal’ probability threshold according to Youden’s J statistic,^26^ and for thresholds corresponding to sensitivities of 80, 85, 90 and 95%. CIs for likelihood ratios were obtained using bootstrap with 10 000 resamples.^27^

## Results

### Participants

**Figure 1:**
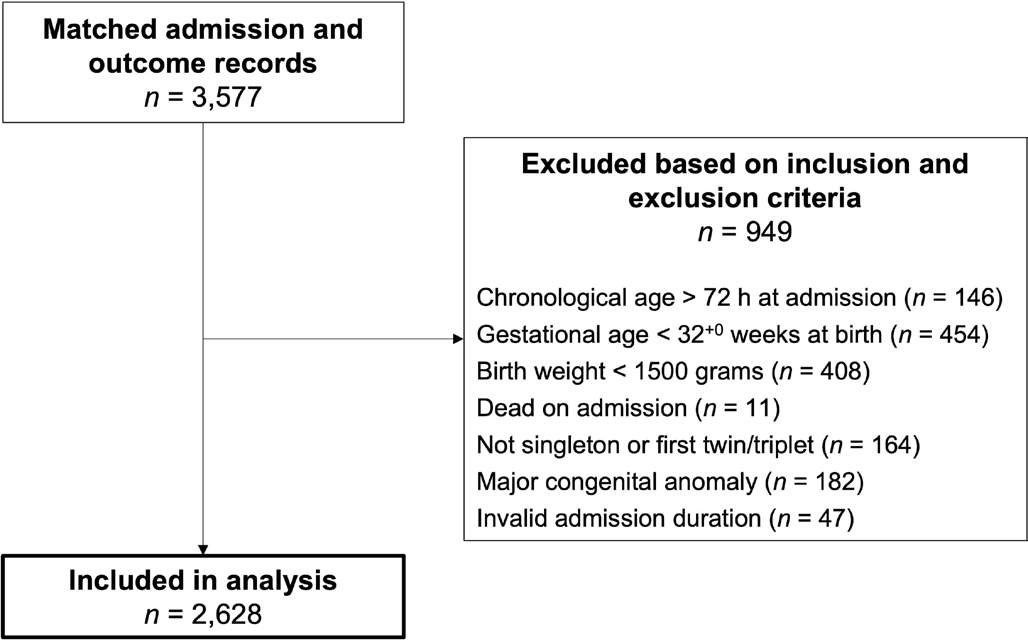
Flow diagram summarising participant inclusion and exclusion. Participants could fulfil multiple inclusion and/or exclusion criteria, therefore, the sum of participants excluded based on each criterion exceeds 949.

Of 3577 neonates with matched admission and outcome records, 2628 (73%) were included (figure 1). Mean gestational age was 38.0 (SD=2.5) weeks, mean birth weight 2890 (SD=700) g, 1141 (43%) received ≥1 antibiotic and 221 (8%) died (table 1). Clinical EOS was diagnosed in 297 neonates (11%, incidence 113 per 1000 admissions).

**Table 1:** Characteristics of the study participants

Characteristics	Overall	No sepsis	Sepsis	P value
n	2628	2331	297	
Admission				
Gestational age (weeks)	38.0 (2.5)	38.0 (2.5)	38.4 (2.3)	0.005
Birth weight (g)	2890 (700)	2880 (720)	2950 (600)	0.07
Sex (%)				0.7
Male	1503 (57)	1338 (57)	165 (56)	
Female	1122 (43)	990 (42)	132 (44)	
Unsure	3 (0.1)	3 (0.1)	0 (0)	
Type of birth (%)				0.03
Singleton	2496 (95)	2205 (95)	291 (98)	
First-born twin	127 (5)	121 (5)	6 (2)	
First-born triplet	2 (<0.1)	2 (<0.1)	0 (0)	
Mode of delivery (%)				0.07
SVD	1889 (72)	1663 (71)	226 (76)	
Elective C-section	136 (5)	124 (5)	12 (4)	
Emergency C-section	561 (21)	510 (22)	51 (17)	
Instrumental	42 (1.6)	34 (1.5)	8 (2.7)	
Postnatal age (%)				<0.001
<2 hours of life	1001 (38)	901 (39)	100 (34)	
2–24 hours of life	1257 (48)	1136 (49)	121 (41)	
24–48 hours of life	235 (9)	181 (8)	54 (18)	
48–72 hours of life	110 (4)	91 (4)	19 (7)	
Outcome				
Antibiotics (%)	1141 (43)	874 (37)	267 (90)	<0.001
Admission duration (days)	2.3 [1.3–4.9]	2.1 [1.2–4.1]	6.0 [3.5–8.8]	<0.001
Death (%)	221 (8)	184 (8)	37 (12)	0.008

Data are presented as mean (SD), n (%) or median [quartile 1 to quartile 3]. P values are from Welch’s two-sample t-test for gestational age and birth weight; the Wilcoxon-Mann-Whitney U test for admission duration; Pearson’s χ^2^ test for postnatal age at admission, antibiotics and death; and Fisher’s exact test for sex, type of birth and mode of delivery. Summary statistics are presented for the observed data only, before multiple imputation of missing values.

C-section, caesarean section; SVD, spontaneous vaginal delivery.

### Missing data

In total, 14 variables had missing values. All variables had <1% missing values except temperature (31%) and birth weight (1.2%). Time since the start of data collection predicted missing temperature (OR 0.96, 95% CI 0.96–0.96, p<0.001) as a limited number of thermometers were available early in the study. Missing temperature was not associated with clinical EOS (OR 0.79, 95% CI 0.60–1.03, p=0.08).

### Model development

From the set of 22 candidate predictors (table 2), eight were excluded due to <5% category prevalence with a non-significant Fisher’s exact test (cyanosis, seizures, fontanelle, colour, abdominal distention, omphalitis, abnormal skin appearance and history of vomiting). Three of the five categories for activity had a prevalence of <5% in either outcome group but Fisher’s exact test indicated a significant difference in the distribution between the two groups (p<0.001). Activity was retained as a predictor and the three smaller categories were collapsed into one ‘other’ group.

**Table 2:** Distributions of candidate predictors in the study cohort

Candidate predictor	Overall	No sepsis	Sepsis	P value
n	2628	2331	297	
Infant risk factors				
Gestational age (weeks)	38.0 [37.0–40.0]	38.0 [37.0–40.0]	38.0 [37.0–40.0]	0.03
Birth weight (g)	2950 [2400–3350]	2900 [2400–3350]	3000 [2600–3350]	0.04
Maternal risk factors (%)				
Maternal fever	14 (0.5)	8 (0.3)	6 (2.0)	0.003
Offensive liquor	163 (6)	131 (6)	32 (11)	0.001
PROM	303 (12)	257 (11)	46 (15)	0.03
Infant clinical features				
Grunting at triage (%)	750 (29)	654 (28)	96 (32)	0.13
Cyanosis at triage* (%)	69 (2.6)	60 (2.6)	9 (3.0)	0.6
Seizures at triage* (%)	14 (0.5)	10 (0.4)	4 (1.3)	0.06
Respiratory rate (breaths/min)	56 [48–68]	56 [48–68]	60 [50–72]	<0.001
Heart rate (beats/min)	138 [126–146]	138 [126–146]	139 [127–150]	0.01
Temperature (°C)	36.5 [36.0–37.0]	36.5 [36.0–36.9]	36.9 [36.2–38.0]	<0.001
Fontanelle* (%)				0.9
Flat	2608 (99)	2312 (99)	296 (100)	
Sunken	10 (0.4)	9 (0.4)	1 (0.3)	
Bulging	10 (0.4)	10 (0.4)	0 (0)	
Activity† (%)				<0.001
Alert	2152 (82)	1933 (83)	219 (74)	
Lethargic	382 (15)	327 (14)	55 (19)	
Irritable	62 (2.4)	45 (1.9)	17 (6)	
Seizures	14 (0.5)	9 (0.4)	5 (1.7)	
Coma	18 (0.7)	17 (0.7)	1 (0.3)	
Nasal flaring (%)	912 (35)	791 (34)	121 (41)	0.02
Chest retractions (%)	986 (38)	848 (36)	138 (46)	<0.001
Grunting (%)	421 (16)	360 (15)	61 (21)	0.03
Work of breathing (%)				<0.001
Normal	1405 (54)	1263 (55)	142 (48)	
Mildly increased	413 (16)	378 (16)	35 (12)	
Moderately increased	614 (24)	529 (23)	85 (29)	
Severely increased	170 (6)	139 (6)	31 (11)	
Colour* (%)				0.1
Pink	2507 (95)	2220 (95)	287 (97)	
Pale	10 (0.4)	7 (0.3)	3 (1.0)	
Blue	62 (2.4)	58 (2.5)	4 (1.3)	
Yellow	49 (1.9)	46 (2.0)	3 (1.0)	
Abdominal distention* (%)	28 (1.1)	26 (1.1)	2 (0.7)	0.8
Omphalitis* (%)	6 (0.2)	4 (0.2)	2 (0.7)	0.1
Abnormal skin* (%)	27 (1.0)	23 (1.0)	4 (1.3)	0.5
Vomiting* (%)				0.3
No	2605 (99)	2309 (99)	296 (100)	
Yellow	7 (0.3)	7 (0.3)	0 (0)	
Bilious	13 (0.5)	13 (0.6)	0 (0)	
Blood stained	3 (0.1)	2 (<0.1)	1 (0.3)	

Data are presented as median [quartile 1 to quartile 3] for continuous predictors or n (%) for categorical predictors. P values are from the Wilcoxon-Mann-Whitney U test for continuous predictors and Fisher’s exact test for categorical predictors. Distributions are presented for the observed data only, before multiple imputation of missing values.

*Eliminated from the final set of candidate predictors due to very skewed distributions.

†The three smallest categories of activity were collapsed into one ‘other’ category for model development.

PROM, prolonged rupture of membranes.

Therefore, 14 candidate predictors were considered for model development. Of these, 12 had a significant univariable association with clinical EOS (table 3). The strongest univariable predictor was maternal fever (OR 6.0, 95% CI 2.1–17.4). Neither birth weight (OR 1.14, 95% CI 0.96–1.35) nor grunting at triage (OR 1.23, 95% CI 0.95–1.59) predicted clinical EOS in univariable models.

**Table 3:** Univariable association between candidate predictors and outcome

Candidate predictor	Coefficient	SE	OR	95% CI	P value
Infant risk factors					
Gestational age (weeks)	0.067	0.026	1.07	1.02–1.12	0.009
Birth weight (kg)	0.131	0.087	1.14	0.96–1.35	0.1
Maternal risk factors					
Maternal fever	1.79	0.544	5.99	2.06–17.4	0.001
Offensive liquor	0.707	0.208	2.03	1.35–3.05	0.001
PROM	0.391	0.173	1.48	1.05–2.08	0.02
Infant clinical features					
Grunting at triage	0.203	0.132	1.23	0.95–1.59	0.1
Respiratory rate (5 breaths/min)	0.093	0.022	1.10	1.05–1.14	<0.001
Heart rate (5 beats/min)	0.047	0.019	1.05	1.01–1.09	0.01
Temperature (°C)	0.886	0.087	2.42	2.04–2.88	<0.001
Activity					
Alert—lethargic	0.395	0.162	1.48	1.08–2.04	0.02
Alert—other	1.05	0.25	2.86	1.75–4.67	<0.001
Nasal flaring	0.290	0.126	1.34	1.04–1.71	0.02
Chest retractions	0.417	0.124	1.52	1.19–1.93	0.001
Grunting	0.346	0.155	1.41	1.04–1.92	0.03
Work of breathing					
Normal—mildly increased	−0.207	0.197	0.81	0.55–1.20	0.3
Normal—moderately increased	0.345	0.146	1.41	1.06–1.88	0.02
Normal—severely increased	0.674	0.217	1.96	1.28–3.00	0.002

Analyses were performed on the complete data after multiple imputation of missing values.

PROM, prolonged rupture of membranes;

Among plausible multivariable models, a model containing eight of the 14 candidate predictors was selected as the optimal model (online supplemental file 2, section 8). Fitting non-linear effects for temperature or birth weight, or allowing for an interaction between birth weight and gestational age, did not improve fit.

### Model specification

The optimal model included eight predictors: temperature at admission, respiratory rate, maternal fever during labour, offensive liquor, prolonged rupture of membranes, activity, chest retractions and grunting (table 4). It can be written as:

$$LP(EOS)=\begin{array}{l} -39.4+0.99\times temperature+0.06\times (respiratoryratedividedby5)+1.44\\ \times maternalfeverduringlabour+0.54\times offensiveliquor+0.36\\ \times prolongedruptureofmembranes+0.59\times lethargy+0.84\\ \times irritability,seizuresorcoma+0.41\times chestretractions+0.18\times grunting \end{array}$$

**Table 4:** Predictors and their pooled regression coefficients and ORs for the optimal model

Candidate predictor	Coefficient	SE	OR	95% CI	P value
Intercept	−39.4	3.52			
Temperature (°C)	0.987	0.095	2.68	2.23–3.23	<0.001
Respiratory rate (5 breaths/min)	0.055	0.026	1.06	1.00–1.11	0.04
Maternal fever	1.44	0.612	4.21	1.27–14.0	0.02
Offensive liquor	0.543	0.228	1.72	1.10–2.69	0.02
PROM	0.360	0.192	1.43	0.98–2.09	0.06
Activity (Alert—lethargic)	0.586	0.184	1.80	1.25–2.58	0.002
Activity (Alert—other)	0.840	0.286	2.32	1.32–4.06	0.003
Chest retractions	0.406	0.172	1.50	1.07–2.10	0.02
Grunting	0.179	0.186	1.20	0.83–1.72	0.3

Analyses were performed on the complete data after multiple imputation of missing values.

PROM, prolonged rupture of membranes.

where *LP*(*EOS*) denotes the linear predictor based on the logit transformation of the probability of clinical EOS. The probability of clinical EOS (*Pr*(*EOS*)) is thus given by the inverse logit function:

$$Pr(EOS)=\frac{e^{LP(EOS)}}{1+e^{LP(EOS)}}$$

### Model performance

**Figure 2:**
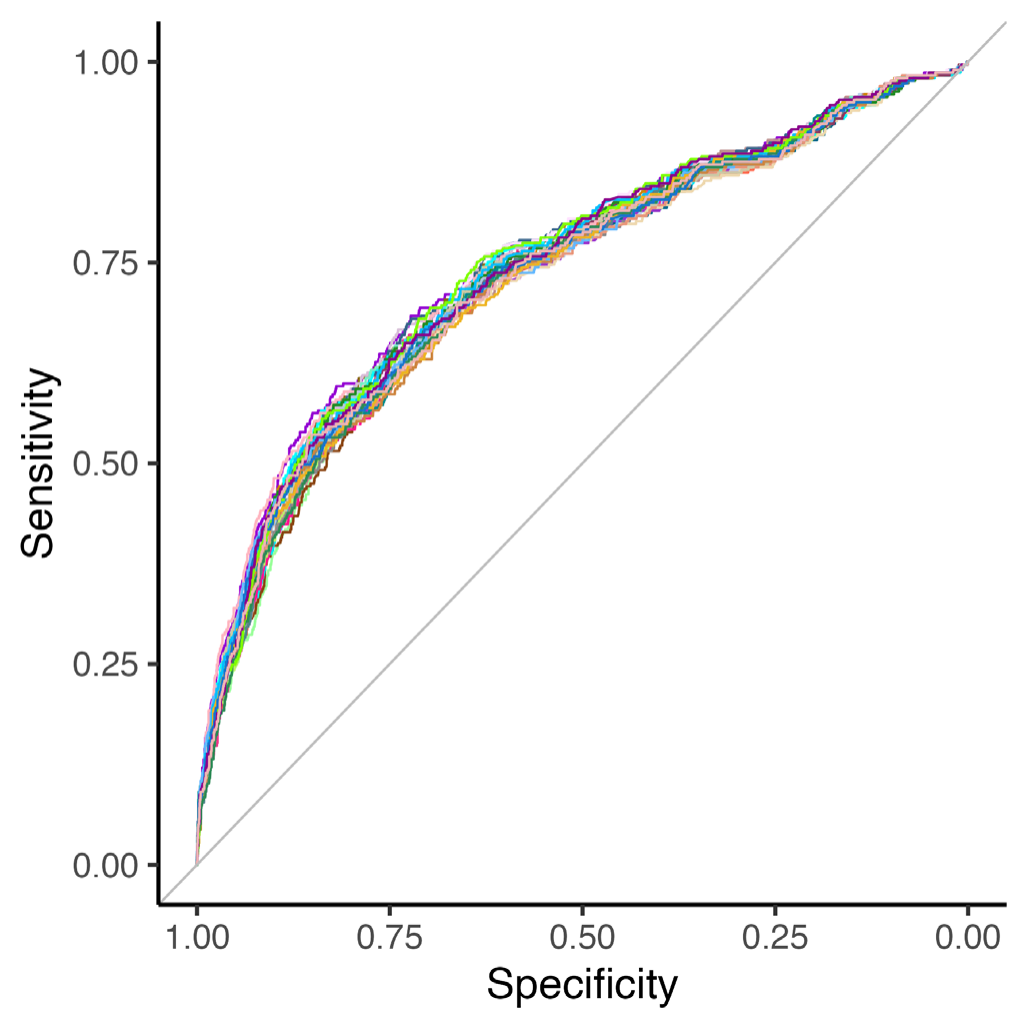
Receiver operating characteristic curve for the optimal model in each of the 40 imputed data sets. Pooled area under the curve (AUC)=0.74 (95% CI 0.70–0.77).

**Figure 3:**
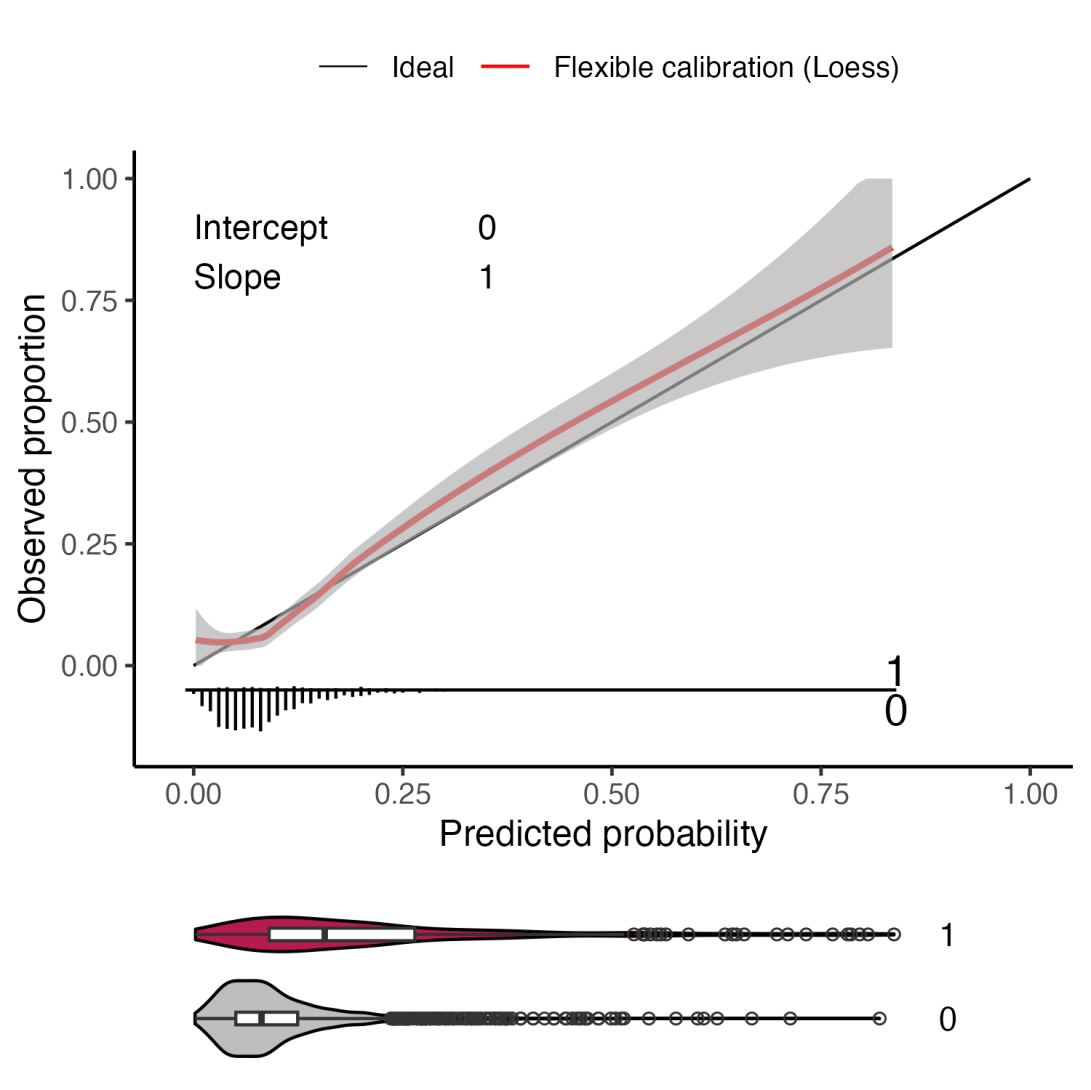
Calibration curve for the optimal model in the single data set used for model selection. A flexible curve with pointwise 95% CIs (shaded region) was fitted using local regression (loess). Calibration intercept=0.00 (95% CI −0.13 to 0.13); calibration slope=1.00 (95% CI 0.85–1.15). At the bottom of the figure, a violin plot shows the distribution of predicted probabilities for neonates with (1) and without (0) sepsis.

The pooled AUC was 0.74 (95% CI 0.70–0.77) (figure 2). The calibration intercept was 0.00 (95% CI −0.13 to 0.13), calibration slope 1.00 (95% CI 0.85–1.15) and the calibration curve remained close to the identity line (figure 3).

The ‘optimal’ classification threshold was 0.12 (ie, 12% predicted probability of clinical EOS) yielding sensitivity 65% (95% CI 59%–70%) and specificity 74% (95% CI 72%–75%) (table 5). For a sensitivity of 95%, the corresponding classification threshold was 0.03 giving sensitivity 95% (95% CI 92%–97%) and specificity 11% (95% CI 10%–13%). Corresponding predictive values and likelihood ratios are shown in table 5.

**Table 5:** Model performance at several classification thresholds of predicted probability

Threshold	Sensitivity	Specificity	PPV	NPV	LR+	LR−
0.121*	65 (59–70)	74 (72–75)	24 (21–27)	94 (93–95)	2.4 (1.6–2.9)	0.5 (0.4–0.6)
0.075	81 (76–85)	44 (42–46)	15 (14–17)	95 (93–96)	1.4 (1.0–1.6)	0.4 (0.4–0.5)
0.067	85 (80–88)	38 (36–40)	15 (13–17)	95 (94–96)	1.4 (1.2–1.6)	0.4 (0.2–0.5)
0.047	90 (86–93)	22 (20–24)	13 (12–14)	95 (92–96)	1.2 (0.9–1.2)	0.4 (0.3–0.6)
0.034	95 (92–97)	11 (10–13)	12 (11–13)	95 (92–97)	1.1 (1.0–1.1)	0.4 (0.3–0.6)

Data are presented for the single data set used for model selection. Numbers in brackets represent the 95% CIs.

*The ‘optimal’ threshold according to Youden’s J statistic.

LR+, positive likelihood ratio; LR−, negative likelihood ratio; NPV, negative predictive value; PPV, positive predictive value;

## Discussion

We developed a clinical prediction model to diagnose clinical EOS that can be applied in LRS. The optimal model included eight predictors: three perinatal risk factors (maternal fever during labour, offensive liquor and prolonged rupture of membranes) and five clinical signs in the neonate (temperature, respiratory rate, activity on neurological examination, chest retractions and grunting). Using a classification threshold for high sensitivity resulted in low specificity in the derivation cohort.

### Interpretation

Incidence of clinical EOS was 113 per 1000 admissions. This is greater than a recent estimate for EOS in low-income and middle-income countries of 31.1 per 1000 live births (95% CI 9–100; I^2^ 99.9%),^28^ but there is marked heterogeneity between relatively few studies worldwide.

Our model shares predictors with existing models for neonatal sepsis.^13^ While several models do not require laboratory tests (some of which have been validated in LRS), data are limited to a few small studies and comparisons are challenging as studies infrequently report global performance measures such as AUC. For example, Weber *et al* developed a score with 14 clinical features to predict neonatal sepsis, meningitis, pneumonia or hypoxaemia in LRS countries.^29^ Validation in the subgroup of 285 neonates aged ≤6 days of life showed a sensitivity of 95% with a specificity of 26% if one or more clinical features were present.^29^

The Kaiser Permanente Early-Onset Sepsis Calculator combines perinatal risk factors with clinical appearance to recommend management based on the estimated probability of EOS in neonates born at ≥34 weeks’ gestation.^30 31^ Meta-analyses suggest its use reduces rates of admission, antibiotic use and use of laboratory tests, without increased mortality (although some authors have voiced concerns about ‘missed’ or delayed diagnoses).^32–35^ All studies in these meta-analyses were from high-income countries.

The Kaiser Permanente Calculator does not require laboratory tests but may be less suited to LRS. First, the baseline incidences of EOS used are lower than in most LRS.^28 30^ Second, the calculator was developed in a population where Group B *Streptococcus* (GBS) is the predominant organism in EOS and where antenatal GBS screening is performed routinely. Finally, descriptors used for clinical presentation (‘clinical illness’, ‘equivocal’ and ‘well appearing’) include interventions such as mechanical ventilation, which are not useful measures of illness in neonatal units where these interventions are unavailable. Two studies have validated the Kaiser Permanente Calculator in middle-income countries with variable results.^36 37^ No studies have validated the calculator in low-income countries or sub-Saharan Africa.

### Implications

Our model includes clinical predictors and risk factors that are simple to identify by any grade of HCP with minimal additional training. Acceptable classification thresholds will vary by clinical context. High sensitivity is important to avoid missing true cases of sepsis, but higher specificity would reduce inappropriate antimicrobial therapy and might be favoured during periods of resource shortages to allow treatment of neonates with the highest probability of EOS.

Our model may be suited to excluding EOS given its low negative likelihood ratio (which represents the change in pretest to post-test odds of having EOS given our model classified a neonate as ‘no EOS’^38^). At a classification threshold of 0.03, the negative likelihood ratio was 0.4 (95% CI 0.3–0.6): a 60% reduction in the odds of EOS for neonates classified as ‘no EOS’. In our cohort, the model had a high negative predictive value (which represents the probability that a neonate does not have EOS if our model classified them as ‘no EOS’^38^). Approximately 300 neonates are admitted each month to SMCH.^39^ With a negative predictive value of 95% and our EOS prevalence of 113 per 1000 admissions, we would expect one or two true cases of EOS to be missed per month.

We would suggest managing neonates classified as having EOS with parenteral antibiotic therapy as per local protocols. Management of neonates classified as ‘no EOS’ would depend on the chosen classification threshold (and resultant negative predictive value) and local HCPs’ and families’ attitudes to risk. Neonates are assessed by the Neotree on admission to the neonatal unit, suggesting they appear unwell to an HCP (nurse, midwife or obstetrician). If classified as ‘no EOS’ by our model, neonates should be observed and investigated for an alternative diagnosis. It may be useful to reapply our model (eg, at 12 hours) to update predictions when the clinical picture has evolved. This is feasible given median admission duration in our cohort was 2.1 (IQR 2.9) days for those without EOS, although further research is required to validate the model in this context.

### Limitations

First, the Neotree collects data at admission and on discharge or death. Neonates admitted for ‘safekeeping’ could have unremarkable clinical appearance and vital signs on admission but develop signs of sepsis later during admission.

Second, very preterm and very low birthweight neonates were not included. Our study focused on stratifying risk of EOS in moderate to late preterm and term neonates, where evidence-based recommendations advising against antibiotics might be more readily observed.

Third, no specific actions were performed to blind outcome assessment. As we performed secondary analysis of data from a quality improvement project, the consultant neonatologist is unlikely to have been biased in their classification of EOS.

Fourth, although blood culture is the gold standard method for diagnosing EOS, erratic supplies of lab reagents meant we could not assess the correlation between positive blood cultures and the consultant neonatologists’ diagnosis of EOS.

Finally, we present model performance in the derivation data, which can be optimistic due to overfitting.^12^

## Conclusions

We developed a prediction model to diagnose clinical EOS using eight predictors. For high sensitivity it achieved low specificity, suggesting it may be suited to excluding EOS to support HCPs’ decisions to withhold antibiotics in non-septic neonates. Our future work will examine (1) external validation; (2) acceptability and feasibility of implementation via the Neotree; and (3) impact of implementation on sepsis-related neonatal morbidity and mortality.

## Data Availability

Data are available upon reasonable request. An open-source, anonymised research database is planned as part of the wider Neotree project. Currently, sharing of deidentified individual participant data will be considered on a case-by-case basis.
